# Using Structured Additive Regression Models to Estimate Risk Factors of Malaria: Analysis of 2010 Malawi Malaria Indicator Survey Data

**DOI:** 10.1371/journal.pone.0101116

**Published:** 2014-07-03

**Authors:** James Chirombo, Rachel Lowe, Lawrence Kazembe

**Affiliations:** 1 Chancellor College, University of Malawi, Zomba, Malawi; 2 UNC Project, Lilongwe, Malawi; 3 Institut Català de Ciències del Clima (IC3), Barcelona, Spain; 4 Abdus Salam International Centre for Theoretical Physics, Trieste, Italy; 5 Department of Statistics and Population Studies, University of Namibia, Windhoek, Namibia; Université Catholique de Louvain, Belgium

## Abstract

**Background:**

After years of implementing Roll Back Malaria (RBM) interventions, the changing landscape of malaria in terms of risk factors and spatial pattern has not been fully investigated. This paper uses the 2010 malaria indicator survey data to investigate if known malaria risk factors remain relevant after many years of interventions.

**Methods:**

We adopted a structured additive logistic regression model that allowed for spatial correlation, to more realistically estimate malaria risk factors. Our model included child and household level covariates, as well as climatic and environmental factors. Continuous variables were modelled by assuming second order random walk priors, while spatial correlation was specified as a Markov random field prior, with fixed effects assigned diffuse priors. Inference was fully Bayesian resulting in an under five malaria risk map for Malawi.

**Results:**

Malaria risk increased with increasing age of the child. With respect to socio-economic factors, the greater the household wealth, the lower the malaria prevalence. A general decline in malaria risk was observed as altitude increased. Minimum temperatures and average total rainfall in the three months preceding the survey did not show a strong association with disease risk.

**Conclusions:**

The structured additive regression model offered a flexible extension to standard regression models by enabling simultaneous modelling of possible nonlinear effects of continuous covariates, spatial correlation and heterogeneity, while estimating usual fixed effects of categorical and continuous observed variables. Our results confirmed that malaria epidemiology is a complex interaction of biotic and abiotic factors, both at the individual, household and community level and that risk factors are still relevant many years after extensive implementation of RBM activities.

## Introduction

Malaria imposes the biggest health burden in Malawi and is one of the leading causes of morbidity and mortality in children under five years of age and pregnant women [1]. It is mainly caused by *Plasmodium falciparum* accounting for 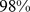 of all malaria cases [2]. About 6 million clinical malaria cases are reported every year and the disease is responsible for about 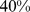 of all hospitalizations of children under the age of five [3]. The disease is endemic to Malawi although there are variations in prevalence across the country [4]. Higher altitude areas with lower temperatures such as Nyika Plateau have lower malaria prevalence than low lying areas with higher temperatures [5]. Transmission takes place throughout the year but peaks during the rainy season from November to April [1].

Malaria transmission is driven by several factors including climatic, geographic, and socio-economic variables [6]. An optimum combination of temperature, humidity and rainfall is required to provide the best conditions for the breeding and development of malaria vectors. Temperature is known to influence the rate of development of the life cycle of the mosquitoes and also the development of malaria parasite. Low temperatures below 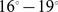
C have the effect of limiting the transmission of *Plasmodium falciparum*. On the other hand, at higher temperatures above 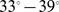
C parasite development ceases [7]. Temperature further dictates the latitudinal and altitudinal ranges of the vector [8]. Nsanje and Chikwawa districts in the Shire River valley for example possess the right combination of environmental and climatic conditions to increase malaria transmission [1].

Variations in malaria risk are also found across the socio-economic spectrum. On the global scale, malaria greatly affects the least developed nations in tropical and sub-tropical regions. Poverty and malaria have been shown to be intimately related [9], [10], with the poorest sub-Saharan countries the worst affected in Africa. Children from rural and less privileged families are more vulnerable to malaria and have a higher risk of developing severe malaria than children from urban areas.

Malawi has been implementing the Roll Back Malaria (RBM) activities such as information, education and communication (IEC) for many years to combat the disease. Insecticide treated nets (ITNs) and now long lasting insecticide nets (LLINs) are the most common interventions in Malawi. Indoor residual spraying (IRS) is another strategy for vector control. ITNs have the potential to reduce malaria transmission when used in control interventions. In Bangladesh, LLIN use was successful in reducing malaria episodes by half and deaths in children by one fifth [11]. On the other hand, distribution of LLINs failed to achieve a reduction in malaria transmission in Zambia and Botswana [12], [13]. Even though the Malawi government and its development partners freely distribute ITNs during periodic mass campaigns, as of 2012 only 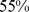 of households in Malawi owned at least one ITN and 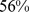 of children under five years of age slept under an ITN the previous night [14]. Spatial disparities exist in the geographical coverage of ITNs [15]. Data from nationwide surveys such as the Malawi Demographic Health Survey have consistently shown variations in proportion of children under the age of five sleeping under a mosquito net.

Malaria prevalence greatly varies across the country as a result of variations in these risk factors. Efforts to spatially analyze malaria prevalence and risk have been made in Malawi [4], [5]. Lack of geo-referenced data that is required for spatial analyses of malaria data has to some extent led to the relatively limited efforts in this area. However recent malaria surveys that have been carried out in Malawi have extensive coverage and collect geographical coordinates to permit the investigation of spatial variability in disease risk. Risk mapping of the disease is crucial in an economically constrained country like Malawi as it enables efficient allocation of scarce resources.

Attempts to map malaria prevalence in Malawi have been made over the years. A risk map generated from point reference data from different sites across the country has been produced [4]. Malaria mapping has also been done at the regional level in northern Malawi using a spatial model applied to hospital case data [5]. More recently, a spatio-temporal statistical model, which maps health facility malaria cases at the district level from 2004–2011 was developed incorporating socio-economic and climatic factors [6]. In order to evaluate the impact of scale up of malaria interventions, the transmission intensity of malaria was mapped for the 10 year period between 2000 and 2010 using a spatio-temporal model [16]. This effort resulted in the production of risk maps at different time points over the decade. However, the model did not consider risk factors or predictors for the disease.

In this study, we investigate if climatic, socio-economic, topographical and environmental risk factors for malaria have remained relevant after many years of implementing interventions such as ITN, IRS and IEC. To achieve this objective, we developed a structured additive regression model, implemented in a Bayesian framework using Markov Chain Monte Carlo (MCMC), to analyze the 2010 Malawi Malaria Indicator Survey (MMIS) data.

## Materials and Methods

### Study area

Malawi is a small country in Southern Africa bordered by Zambia, Mozambique and Tanzania. The country experiences rainfall mainly between November and April and malaria transmission peaks shortly after this period. The first MMIS was done in 2010 and will be conducted every two years to provide data on malaria prevalence in line with the National Malaria Control Programme (NMCP) strategic plan. Malawi is implementing malaria control interventions under the RBM partnership such as ITNs/LLINs and IRS which are supported by Global Fund, the President's Malaria Initiative and several other organizations. The NMCP coordinates the intervention and control activities in Malawi.

### Data sources

The malaria data used for the analysis were obtained from the 2010 MMIS which was the first country wide malaria prevalence survey to be conducted in Malawi. The survey took place during March-April 2010, at the end of the rainy season in Malawi. A total of 3,500 households were selected for data collection. Sample size determination used initial assumptions from the Malaria Alert Centre household survey of 2007 [2]. A two stage cluster sampling was used to select the households. The first stage selected 140 enumeration areas (EAs) of which 96 were from rural areas and 44 from urban centres. The EAs were selected proportionately to the regional population. At the second stage, 25 households per EA were selected. Data were collected from all but three districts namely Mwanza, Neno and Likoma.

In the selected households, children were tested for malaria using rapid diagnostic tests (RDT) to determine prevalence. Women were asked questions with regards to their knowledge of the disease using a face-to-face questionnaire. Variables collected were age of the child, ITN use, altitude, wealth and sex of the child. The wealth index for each household was computed using data on the household's ownership of selected assets and sanitation facilities (such as televisions, bicycles, type of drinking water source and type of toilet facility). All households were then placed into five wealth quintiles with 1 being the poorest and 5 the richest. ITN usage was determined by asking parents about ownership and usage of the net the night before the survey. The location (rural or urban) of the households and their region within the country were recorded. Latitude and longitude for each household were also collected by GPS. The MMIS datasets can be obtained upon request from MEASURE DHS [17].

Climate data for each EA were obtained from the Department of Meteorological Services and Climate Change in Malawi [18], collected through the network of over 20 weather stations across the country. Mean climatic variables for rainfall (mm/day) and minimum temperature (
C) averaged over the three months preceding the survey (January-March) were calculated and used in the analysis.

### Model formulation

Suppose 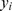 is the malaria status of a child 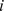 such that a positive malaria test is recorded as 1, or 0 otherwise. Then, binary response data is generated, which follows a Bernoulli distribution
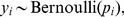
(1)where 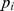 is the probability of a positive test. With an appropriate link function, the risk of malaria disease can be associated with explanatory variables using a generalized linear model (GLM) framework. GLMs are a flexible alternative to ordinary linear regression, that allow for non-normal response variables [19].

The GLM can be specified with linear predictor 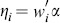, where 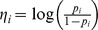 is the logit link function and 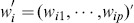 is a matrix of explanatory variables. An ordinary logistic regression is then specified as follows:
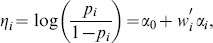
(2)where 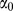 is the intercept, 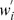 is a vector of covariates and 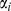 is a vector of regression coefficients.

A limitation to standard GLMs is that they assume independent (or at least uncorrelated) observations. However, this assumption is not always met as sometimes observations exhibit spatial and/or temporal dependence. This needs to be incorporated in models in order to provide a more accurate estimation and prediction of the response variable. The linear predictor, by taking into account the spatial autocorrelation, can be expanded as follows
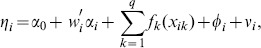
(3)where 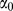 is the intercept, 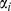 is the parameter corresponding to the categorical fixed variables 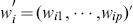 (e.g. wealth index, age category, location, bed net use) and 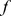 is an appropriate smoothing function of continuous covariates, 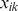 (rainfall, minimum temperature, altitude). Spatially unstructured random effects, 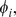 capture the unobserved spatial heterogeneity and overdispersion at each location such as immunity to malaria while spatially structured random effects, 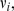 allow for spatial autocorrelation and clustering, for example variation in access to interventions such as ITNs among the communities.

Equation 3 gives rise to a class of models known as structured additive regression (STAR) models. Generalized additive models (GAM) [20], generalized additive mixed models (GAMMs) [21] and geoadditive models [22] are special cases of the STAR models. All of these models make use of smooth functions to model covariate effects on the response variable. These models are increasingly being applied to model health impacts and outcomes such as spatial variation of HIV infections and effects of climate on malaria across Africa [23]–[29].

#### Prior assumptions

The implementation of the model follows a Bayesian approach. In Bayesian analysis, all the regression coefficients and the smooth functions 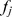 are considered as random variables and are assigned prior distributions. Without any prior knowledge, the coefficients 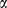 of the continuous covariates are assigned diffuse priors, i.e.
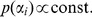
(4)


The unknown smooth functions 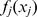 are assigned Bayesian penalized splines priors [30]. The functions are assumed to be approximated by a polynomial of degree 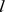 which is defined over a set of equally spaced knots of the form 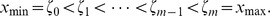 The spline is expressed as a linear combination of B-spline basis functions. This approach is similar to fitting second order random walk priors of the form 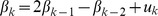 with Gaussian errors, 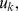 assigned to the smooth terms. The spatial random terms are also fitted as splines, particularly as a two-dimensional tensor product. The unknown 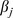 are assigned priors of the general form
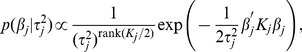
(5)where 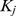 is the penalty matrix and 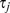 is the variance parameter that controls the tradeoff between flexibility and smoothness. The 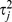 is assigned non-informative dispersed inverse Gamma priors 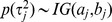 [31] where
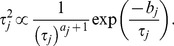
(6)To capture the spatial effects we assumed stationary Gaussian process with zero mean and variance 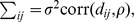 where 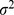 is the sill, and 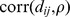 is the spatial correlation. The spatial correlation is considered a function of distance, 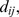 between the spatial locations 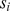 and 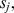 under isotropic assumptions. Usually the exponential correlation function is assumed such that 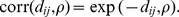 The parameter 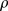 measures how fast the correlation decays as the distance between the locations increases [32]. Bayesian inference was done using MCMC simulation based on the posterior distribution 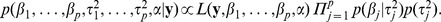


#### Model implementation

In order to assess factors that are associated with the probability of an under five child testing positive for malaria, different models were fitted as follows:
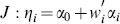





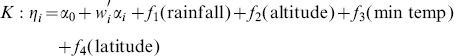





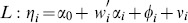





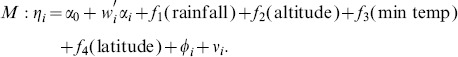
In the fixed effects model, 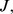 categorical and continuous variables were fitted linearly in the usual GLM framework. In these models, 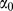 is the intercept and 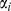 is the vector of coefficients of the categorical variables, 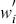. The second model, 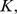 includes smooth functions of the 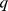 continuous covariates 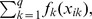 such as rainfall and altitude, to assess the importance of possible non-linear associations. In model 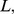 random effects of location were included, together with all other covariates, fitted as fixed effects. Lastly, model 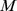 included categorical variables fitted as fixed effects, continuous covariates fitted as smooth functions to account for non-linearity, and spatial random effects.

Bivariate tests were carried out in order to determine which variables to include in the models. Initial descriptive analysis was done using cross tabulations and assessed using the Chi-square test to investigate the relationship between the outcome of the malaria test and several categorical variables at the 95% confidence level (CI).

In running the MCMC algorithm, 10 000 iterations were made with a burn in of 1000 and a thinning parameter of 50. To ensure that the choice of the priors in the Bayesian analysis did not influence the results, a sensitivity analysis was performed by running the chosen model several times, changing the prior parameters at each run and then comparing the observed changes in the estimates. The default gamma prior with hyper-parameters equal to (*a* = 0.001,*b* = 0.001) was changed and the model was run 3 times with the new priors (*a* = 0.00001,*b* = 0.00001), (*a* = 0.0005,*b* = 0.0005) and (*a* = 1,*b* = 0.005). The Deviance Information Criterion (DIC) [33] was used to compare the fitted models J, K, L and M (the smaller the DIC, the better the model). Convergence was assessed through trace plots. Analyses were performed using the free software BayesX [34] in a full Bayesian approach using MCMC. The R statistical software [35] and BayesX package [36] in R were also used to analyze and visualize results.

## Results

The DIC values for the four models based on one set of priors were compared. Model J had DIC of 2193.23, model K had DIC of 2025.85. The DIC values for models L and M were 1927.35 and 1894.35 respectively. Model M, combining categorical variables, smooth functions of continuous variables and the spatial random effects explained childhood malaria risk better than the other models. Therefore, this model was selected for further analysis since it had the lowest DIC. Model M was then subjected to changes in prior hyper-parameters. The model was found not to be sensitive to the changes, indicating that it was an appropriate model to use.

### Observed malaria risk in Malawi

**Figure 1:**
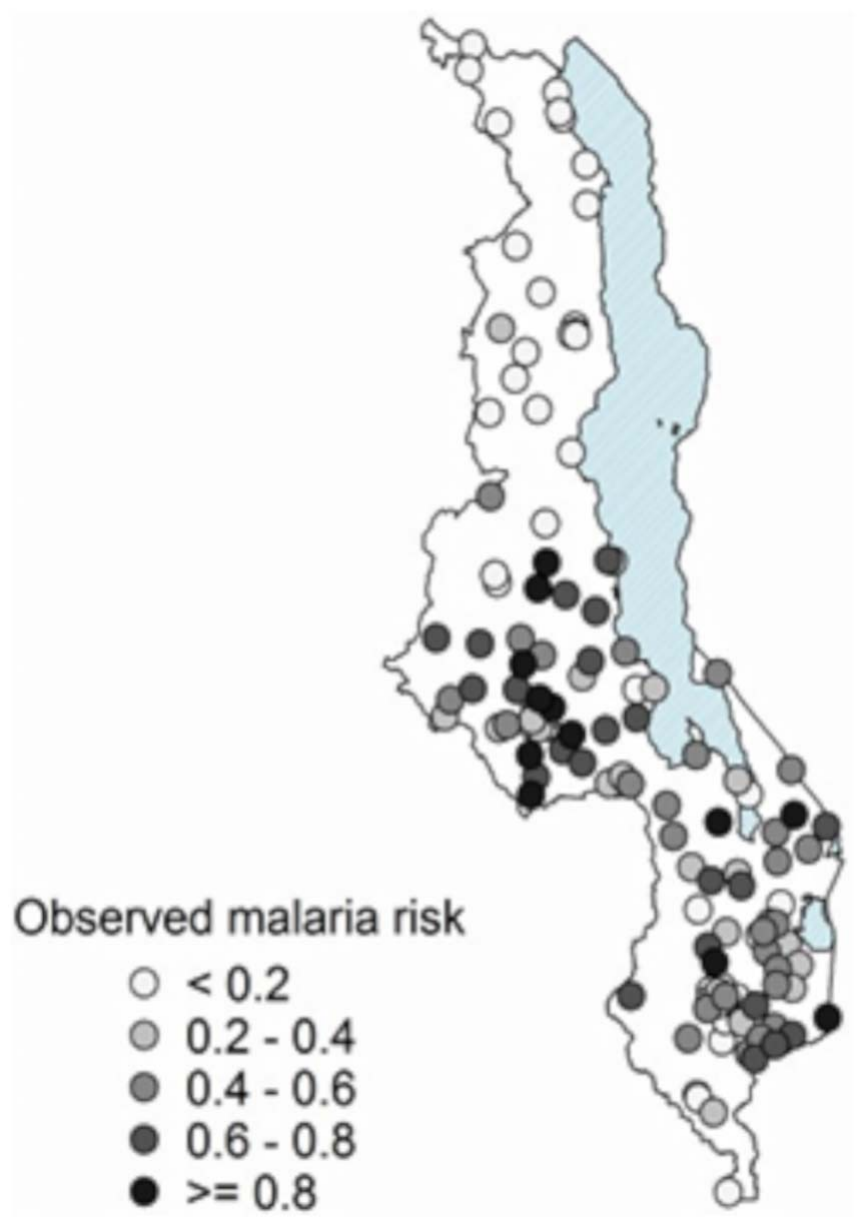
Observed malaria risk in children under five. Observed malaria risk in children under five years at the 140 EAs across Malawi.

Figure 1 shows malaria prevalence per sampled EA across Malawi. It clearly shows that the central and southern regions registered higher malaria risk than the northern region during the survey period. Ntchisi district in the central region registered the highest malaria risk. Table 1 provides a summary of the number of children examined, those testing positive and the calculated malaria risk against data points/EAs per district.

**Table 1:** Under five malaria prevalence by district.

District	No. examined	No. positive	Observed risk	Data points 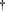
Chitipa	42	1	0.02	3
Karonga	66	6	0.09	4
Nkhata Bay	29	3	0.10	1
Rumphi	32	3	0.09	2
Mzimba	129	14	0.11	11
Kasungu	121	78	0.64	6
Nkhotakota	36	15	0.42	3
Ntchisi	22	17	0.77	1
Dowa	59	31	0.53	4
Salima	67	30	0.45	5
Likoma	NA	NA	NA	NA
Lilongwe	370	159	0.43	24
Mchinji	68	35	0.51	3
Dedza	90	41	0.46	7
Ntcheu	41	19	0.46	3
Mangochi	132	74	0.56	8
Machinga	64	29	0.45	5
Zomba	117	43	0.37	8
Chiradzulu	33	16	0.48	2
Blantyre	208	53	0.25	18
Thyolo	86	34	0.40	5
Mulanje	95	59	0.62	5
Phalombe	68	35	0.51	3
Chikwawa	55	19	0.35	4
Nsanje	31	5	0.16	2
Balaka	33	15	0.45	3
Mwanza	NA	NA	NA	NA
Neno	NA	NA	NA	NA

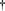
Data points are EAs.

#### Bivariate association between malaria prevalence and risk factors

Table 2 shows the association between malaria risk and selected categorical variables. At the 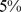 level, a statistically significant association between age of a child, wealth status of a household and malaria status was found 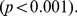 Use of ITN, and place of residence, whether rural or urban and region (north, centre or south) were also significantly associated with malaria status 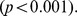 However, gender of a child did not show a statistically significant association with malaria risk 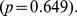


**Table 2:** Association between malaria risk and selected categorical variables.

	Malaria		
Variable	Yes (%)	No (%)	p-value
**Age group (yrs)**			<0.001
0–1	68(25.4)	200(74.6)	
1–2	150(36.2)	264(63.8)	
2–3	186(44.5)	232(55.5)	
3–4	164(46.5)	189(53.5)	
4–5	149(45.0)	182(55.0)	
**ITN**			0.002
Yes	586(39.0)	916(61)	
No	275(46.5)	316(53.5)	
**Sex**			0.649
Male	435(41.6)	610(58.4)	
Female	426(40.6)	622(59.4)	
**Location**			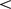 0.001
Urban	91(16.4)	464(83.6)	
Rural	770(50.1)	768(49.9)	
**Region**			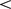 0.001
South	382(41.43)	540(58.57)	
Centre	425(48.63)	449(51.37)	
North	54 (18.12)	244(81.88)	
**Wealth index**			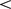 0.001
Poorest	297(59.0)	206(41.0)	
Poorer	161(56.3)	125(43.7)	
Medium	125(43.7)	224(53.0)	
Richer	124(31.4)	271(68.6)	
Richest	80(16.5)	406(83.5)	

**Figure 2:**
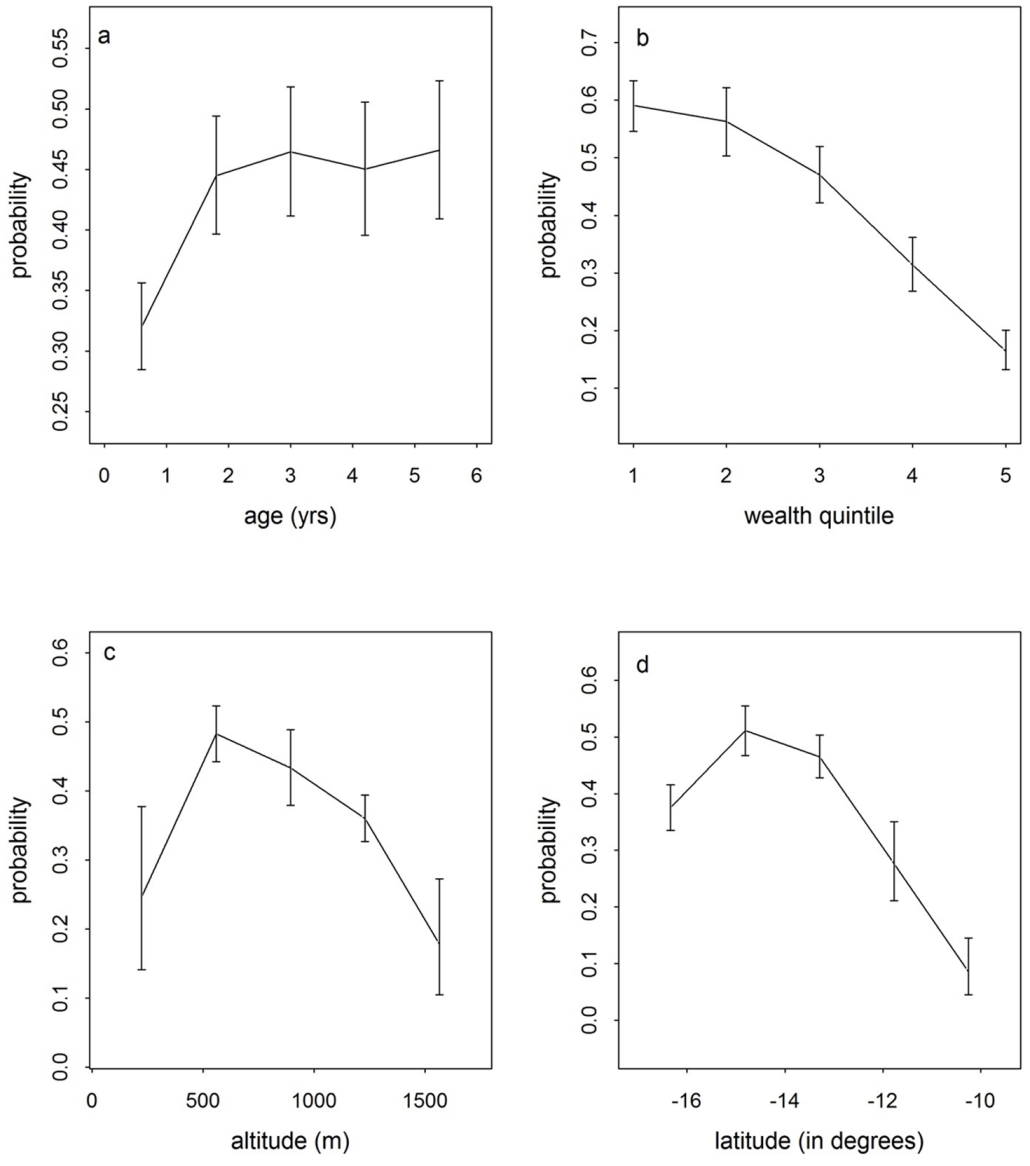
Variation in probability of malaria in children aged less than 5 years with potential risk factors. Variation in probability of malaria in children aged less than 5 years with (a) age (b) wealth index (c) altitude (d) latitude. The vertical bars are 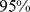 CI.

Figure 2 shows how the risk of malaria varied with changes in different variables. In Figure 2a, the risk of malaria as indicated by the probability of a positive malaria test generally increased as the age approached five with children less than one year of age having the lowest risk of 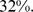 The risk then rose sharply to 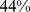 by the time the child reached two years. Beyond this age, the risk remained relatively constant. Fig 2b shows how the risk of malaria dropped as the wealth of a household improved. Children from the poorest households (quintile 1) were at the highest risk (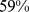) compared to 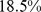 for children from the richest households (quintile 5). It can also be seen from Figure 2c that there was some non linear relationship between malaria risk and altitude. The highest risk was observed at about 600 m above sea level and then dropped with increasing altitude reaching 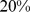 at 1500 m. Lastly, it was observed that the disease risk dropped when moving from north to south of the country (Figure 2d). The lowest risk of 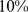 was recorded in the North at around 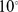
S latitude and the highest probabilities were observed in the central region of Malawi indicated by the mid lying altitudes around 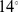
S. In this area, the risk goes up to 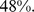


#### Effect of categorical variables on malaria risk

Odds ratios from the best fitting model showing the relationship between the categorical variables and the risk of the disease are shown in Table 3. Children aged between 1–2 years had 2 times higher odds of testing positive than those less than 1 year old (adjusted OR = 2.32, CI: 1.53, 3.51). Age groups 3–4 and 4–5 years both had 5 times higher odds of having the disease (adjusted OR = 5.20, CI: 3.37, 8.02 and adjusted OR = 4.64, CI: 2.99, 7.21, respectively).

**Table 3:** Parameter estimates and 95% credible intervals for the categorical variables of the chosen model.

	Parameter estimates		
Explanatory Variable	Odds Ratio (OR)	2.5% Quantile	97.5% Quantile
**Intercept**	0.17	0.08	0.35
**Age group (yrs)**			
0–1	1.00		
1–2	2.32	1.53	3.51
2–3	3.60	2.37	5.45
3–4	5.20	3.37	8.02
4–5	4.64	2.99	7.21
**ITN**			
Yes	0.57	0.43	0.76
No	1.00		
**Location**			
Rural	4.13	2.31	7.38
Urban	1.00		
**Region**			
South	1.00		
Centre	1.48	0.90	2.42
North	0.15	0.07	0.32
**Wealth index**			
Poorest	1.00		
Poorer	1.10	0.76	1.60
Medium	0.66	0.45	0.96
Richer	0.42	0.28	0.64
Richest	0.22	0.14	0.37

Children from rural areas had 4 times higher odds of contracting malaria than their urban counterparts (adjusted OR = 4.13, CI: 2.31, 7.38). The odds of malaria infection steadily dropped as wealth increased. Children from medium income households were found to have 34% lower odds of malaria compared to those from the poorest households (adjusted OR = 0.66, CI: 0.45, 0.96). The odds further dropped as the children in the richest households had 78% lower odds of parasitaemia (adjusted OR = 0.22, CI: 0.14, 0.37). Children from the central region had 48% greater odds of testing positive for malaria (adjusted OR = 1.48,CI: 0.90, 2.42) than those from the south. The use of bed nets as an intervention yielded positive results as children sleeping under an ITN had 43% lower odds of contracting malaria (adjusted OR = 0.57, CI: 0.43, 0.76) compared to those not sleeping under ITN.

#### Effect of continuous covariates on malaria risk

**Figure 3:**
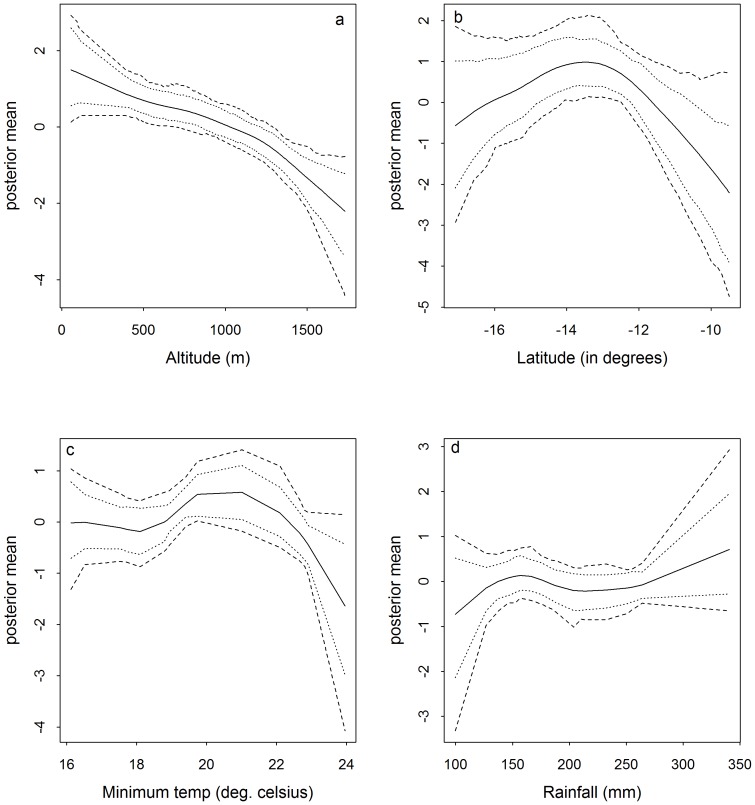
Non linear effect of different continuous covariates on malaria risk (a) altitude (b) latitude (c) minimum temperature (d) rainfall. Plots based on model estimates from the multivariate spatial model with splines (model M). The inner and outer dotted lines are the 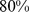 and 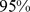 CI respectively. The solid middle line is the posterior mean.

The possible nonlinear effects of the continuous covariates after accounting for other variables are presented in Figure 3 together with 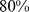 and 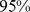 credible intervals. Figure 3a shows a steady drop in malaria risk with increasing altitude. The lowest risk is observed at altitudes above 1500 m above sea level. On the other hand, latitude in Figure 3b does not show an association with the risk as indicated by the CI in the figure. Figure 3c shows that from average minimum temperatures of 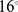
C to 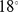
C, risk remains relatively constant. Malaria risk then increased slightly as temperature approached 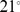
C. Despite these changes in risk, minimum temperature overall was not significantly associated with malaria. Similarly rainfall was not significantly associated with the disease risk as shown in Figure 3d. Malaria risk was lower for average monthly rainfall below 150 mm but increased slightly from 260 mm.

#### Malaria risk map

**Figure 4:**
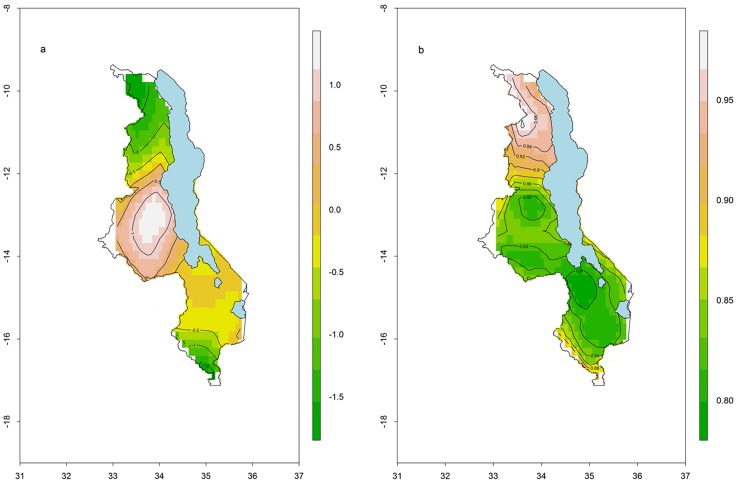
Risk map of malaria in children less than 5 years. (a) Predictive risk map of malaria in children less than 5 years (b) Standard errors associated with the risk map. Green (brown) colours represent lower (higher) standard errors.

Using climatic and environmental variables only, we generated a malaria risk map for Malawi (Figure 4a). The risk map shows that, in general, the central region had the highest risk followed by the southern region. In the northern region, the risk was lower due to the cooler climate in this part of the country, as a result of the mountainous terrain. The southern part of Malawi showed lower than expected risk which could be due to undersampling. The districts of Mwanza and Neno were not sampled and coupled with the low sampling density in Nsanje, this area was not well represented. Figure 4b shows a map of standard errors, indicating that the highest errors are found in the north, compared to the rest of the country. Standard errors were greatest in areas with the lowest sampling density.

## Discussion

We analyzed the data from the first nationwide malaria survey in Malawi to estimate the changes in risk factors of the disease in children aged under five in the face of ongoing interventions. The MMIS provided data on the malaria status, intervention activities, socio-economic status of households and some geographical variables such as location.

The analysis showed that the risk of malaria increases as the child approaches the age of five. The lower risk for children aged between 0–1 years could be explained by the presence of maternal immunity in the infant during the first six months. The 2012 MMIS also showed a similar trend of increasing malaria prevalence with age. For instance RDT results showed a prevalence of 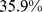 in children 6–8 months of age steadily increasing to 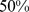 among the 4–5 year age group [14]. During the first 6 months of life, the protective effect of maternal immunity helps prevent malaria attacks [37]. This observed increase in prevalence may be due to some behavioral characteristics at the household level. During the early part of their lives, children tend to be well taken care of and thus protected from many diseases including malaria. However, this changes when they grow older. In Nigeria, a study found that sleeping under an ITN was associated with the age of a child such that children aged less than 1 year were two times more likely to sleep under an ITN than 4 year olds [38].

Similar country wide malaria surveys in Angola and Zambia have shown a significant association between sleeping under ITN and reduction in malaria prevalence among children [39], [40]. However, even though ITNs are freely distributed in Malawi, it has been shown that inequalities to access remain with the poorest missing out on the ITNs [41]. This could be one of the contributing factors to high malaria prevalence among the poorest households. A study in Afghanistan found that families from the richest wealth quintiles were 4.5 times more likely to purchase ITNs than families from the two lower quintiles [42].

The risk map produced showed lowest risk in the northern part of Malawi compared to the other regions. This region constitutes vast mountainous areas, such as the Nyika Plateau with a cooler climate that may discourage vector reproduction and activity. The central region is covered by large portions of inland plain land in Kasungu and Lilongwe and low lying areas along the lake that may offer better conditions for vectors. Very low altitude areas less than 500 m showed lower risk, which could be due to very high temperatures not suitable for vector development and malaria transmission. Temperatures above 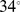
C generally have a negative impact on the survival of parasites. The model also showed lack of significant association between malaria and the climatic variables of rainfall and minimum temperature. A similar lack of association between climatic and environmental variables including rainfall with malaria was observed in Zambia [40].

Adaptation of standard GLMs to allow modelling of possible nonlinear relationships between continuous covariates and the response, in addition to taking into consideration the inherent spatial correlation in the data, leads to more accurate estimates of the risk factors of malaria. Models fitted without taking into account the spatial structure were found to be less adequate when compared with spatial models. Environmental, topographical and climatic variables are usually associated with malaria in the malaria endemic zones, including Malawi, and accounting for these variables in the model leads to more accurate parameter estimates.

One limitation is that the MMIS was carried out during the months with the highest malaria transmission and this restricts the applicability of the malaria risk maps to this time of the year. The lower sampling density in some districts also makes prediction of malaria in unobserved locations in those areas a challenge. For example, the survey did not cover some districts (Mwanza,Neno and Likoma) thus distorting the results to some extent especially in the southern Malawi where Mwanza and Neno are located. The quality of the data is also affected by the possible misclassification of malaria cases.

Despite limitations, the analysis and risk maps indicate that the various risk factors especially geographical such as location of residence (urban or rural) and region remain significant after years of coordinated malaria response under the RBM framework. The NMCP, in collaboration with local authorities has a big task in containing the risk factors within the districts. The NMCP can then extend its reach by coordinating with authorities in neighbouring countries in the fight against the disease. It has been shown that malaria in the border areas is a problem. In Zambia, a study revealed persistent hotspots identified along the Malawi border [13]. The fight against malaria can be greatly improved if both in country and across country interventions work in harmony.

The 2010 MMIS acts as a baseline upon which subsequent surveys will be built. It is crucial to monitor trends in malaria risk among children and to continually explore the complex relationships between parasitaemia risk and environmental, climatic and socio-economic factors. This will be possible since each round of the MMIS will cover the same locations thus making it possible to monitor under five malaria risk over a long period of time. Furthermore, effective control measures of under five malaria at household level in Malawi should start with proper mapping of the disease risk. It is only after understanding the distribution of malaria in Malawi that resources can be prudently allocated to deal with the problem.

## Conclusion

This research provides an empirical risk map that can be used for intervention activities by identifying areas that are likely to have higher risks and hence require special attention. Since the analysis is based on the first country representative survey, the maps produced are the most credible and reliable to date for use in control initiatives. These results, coupled with expert opinion, which is widely used in the absence of empirically produced maps, can lead to a better understanding of the spatial distribution of malaria and hence more targeted interventions in the fight against the disease in young children.
